# Druggable targets and therapeutic development for COVID-19

**DOI:** 10.3389/fchem.2022.963701

**Published:** 2022-10-05

**Authors:** Xiaohua Duan, Lauretta A. Lacko, Shuibing Chen

**Affiliations:** Department of Surgery, Weill Cornell Medicine, New York, NY, United States

**Keywords:** SARS-CoV-2, main protease, papain-like protease, RNA-dependent RNA polymerase, neutralizing antibodies, host factors

## Abstract

Coronavirus disease (COVID-19), which is caused by SARS-CoV-2, is the biggest challenge to the global public health and economy in recent years. Until now, only limited therapeutic regimens have been available for COVID-19 patients, sparking unprecedented efforts to study coronavirus biology. The genome of SARS-CoV-2 encodes 16 non-structural, four structural, and nine accessory proteins, which mediate the viral life cycle, including viral entry, RNA replication and transcription, virion assembly and release. These processes depend on the interactions between viral polypeptides and host proteins, both of which could be potential therapeutic targets for COVID-19. Here, we will discuss the potential medicinal value of essential proteins of SARS-CoV-2 and key host factors. We summarize the most updated therapeutic interventions for COVID-19 patients, including those approved clinically or in clinical trials.

## Introduction

In the past 2 decades, there have been three coronavirus outbreaks, severe acute respiratory syndrome (SARS) in 2003, Middle East respiratory syndrome (MERS) in 2012, and coronavirus disease 2019 (COVID-19) in 2019. For COVID-19, it was identified that the novel coronavirus SARS-CoV-2 was the causative pathogen. SARS-CoV-2, a new member of the genus Betacoronavirus, was most closely related to three bat coronaviruses, BANAL-52, BANAL-103, and BANAL-236, of which the animal reservoir were Laotian R. malayanus, R. pusillus, and R. marshalli, respectively. Notably, BANAL-52 has the highest nucleotide conservation in the receptor-binding domain (RBD) and N-terminal domain (NTD) of the S1 domain in the spike protein (Temmam et al., 2022). It was evidenced that spike (S) protein of SARS-CoV-2 could mediate viral entry through binding to angiotensin converting enzyme 2 (ACE2) (Hoffmann et al., 2020b; Zhou P. et al., 2020). S protein has two subunits, S1 and S2. The 319–529 amino acid peptide of S1 was identified as the ACE2 binding domain, which is the target of neutralizing antibodies (Shang et al., 2020b). Compared with SARS-CoV, the binding domain of SARS-CoV-2 has stronger affinity to human angiotensin-converting enzyme 2 (hACE2). However, there are several additional receptors shown to mediate virus entry, such as CD147, Neuropilin-1, and Dipeptidyl peptidase 4 (DPP4) (Masre et al., 2021). Proteolytic processing of the SARS-CoV-2 S protein is required for activation. (Shang et al., 2020b). Host proteases, including transmembrane protease serine protease 2 (TMPRSS2), cathepsin L, and furin can cleave S protein and facilitate the entry of SARS-CoV-2 (Shang et al., 2020a; Ou et al., 2020). After the RNA genome of SARS-CoV-2 is released into the host cell, 16 non-structural proteins (nsp), four structural proteins, and several accessory proteins are transcribed and translated. The nsps are encoded by two open reading frames, ORF1a and ORF1b, which mediate the viral replication. The structural proteins include S, envelope (E), membrane (M) and nucleocapsid (N). The accessory proteins, which have not been well studied until now, are thought to play a critical role in SARS-CoV-2 pathogenicity and the regulation of the host immune response (Michel et al., 2020). Overall, every process during the viral life cycle relies heavily on the interactions between viral proteins and host factors. Each of these molecular proteins can be targeted for anti-SARS-CoV-2 drug development. In the current review, we will discuss the druggable targets and their potential therapeutic development.

The pathogenesis of most patients with COVID-19 is asymptomatic and mild, however, some patients will develop severe COVID-19 and even respiratory failure. At the initial stage, viral particles invade epithelial cells in the nasopharynx, where they replicate, migrate down to the airway, and then infect alveolar epithelial cells. Compared with other respiratory viruses, the inflammatory response to SARS-CoV-2 infection is significantly different. A delayed interferon response and high expression of IL-6 were defined as the features of COVID-19 (Blanco-Melo et al., 2020). Notably, the severe COVID-19 and death cases are mainly caused by acute respiratory distress syndrome (ARDS), which is a consequence of the cytokine storm. (Mehta et al., 2020; Chen et al., 2021). Here, we will also discuss the current drug development in targeting cytokine storm and ARDS.

## Drug development against targets of SARS-CoV-2

### Non-structural proteins as drug targets

**FIGURE 1:**
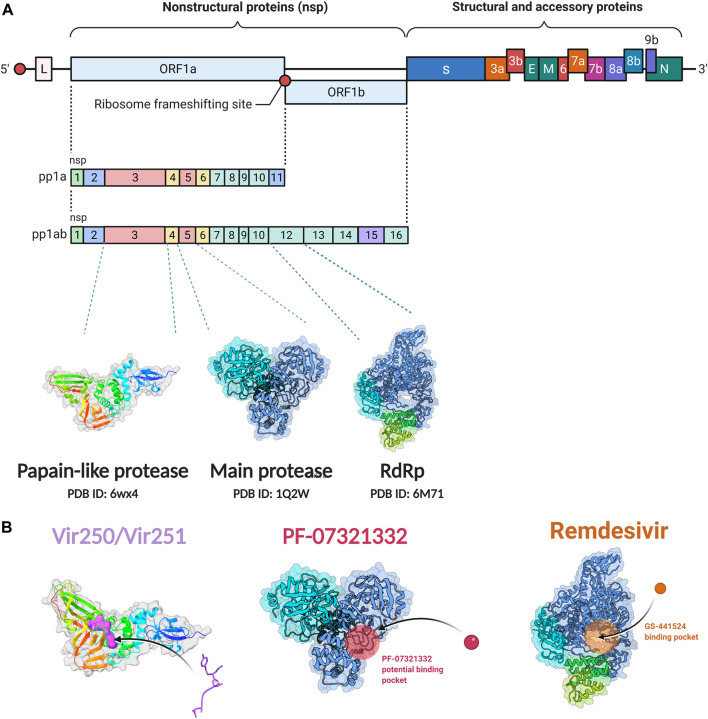
**Non-structural protein as targets for antiviral drug development. (A)** Genome organization of SARS-CoV-2. **(B)** The crystal structure of the non-structural proteins and their inhibitors. The crystal structure of VIR250 and VIR251in complex with PLpro of SARS-CoV-2; the crystal structure of Mpro and its binding pocket of PF-07321332; the crystal structure of RdRp and its binding pocket of remdsivir. The crystal structures were obtained from RCSB Protein Data Bank. Figure was generated by BioRender.

SARS-CoV-2 is a single stranded and positive sense RNA virus (Marra et al., 2003; Chen Y. et al., 2020). The ORF1a and ORF1b in the RNA genome encode two big polypeptides with a ribosomal frameshift, pp1a and pp1ab, which can be cleaved into 16 nsps by nsp3, papain-like protease (PLpro) and nsp5, main protease (Mpro) (Figure 1A). Another important nsp is nsp12, RNA-dependent RNA polymerase (RdRp), which is main component of RNA replication machinery catalyzing the synthesis of RNA (Figure 1A). Due to their critical functions, PLpro, Mpro, and RdRp have been considered as the major drug targets for anti-viral drug development.

#### PLpro and inhibitors

The PLpro of SARS-CoV-2 is an essential coronavirus enzyme encoded by nsp3 (Figure 1A), which cleaves the viral polyprotein to generate a functional replicase complex (Harcourt et al., 2004). PLpro also exhibits deubiquitinating activity on host proteins against host antiviral immune responses (Barretto et al., 2005; Shin et al., 2020). It has been well reported that type I interferons (IFNs) play a key role in the antivirus response through activating the expression of interferon-stimulated genes (ISGs), which could induce the antiviral states of host cells (McNab et al., 2015). Moreover, Type I IFNs can activate both adaptive and innate immune responses by affecting the functions of myeloid cells, B cells, T cells, and NK cells (McNab et al., 2015). For patients with severe COVID-19, impaired type I IFNs response to SARS-CoV-2 infection have been observed (Hadjadj et al., 2020; Lei et al., 2020). SARS-CoV-2-PLpro can remove interferon stimulated gene 15 protein ubiquitylation, and may attenuate host type I IFNs response and promote viral escaping the immune surveillance. (Shin et al., 2020; Osipiuk et al., 2021). Overall, SARS-CoV-2-PLpro is a critical candidate for drug target to inhibit SARS-CoV-2 infection and activate host antiviral immune response.

Several drug screens have been performed against SARS-CoV-2-PLpro, but very few drug candidates have been found, and no clinical trials are currently under evaluation. The first two inhibitors, VIR250 and VIR251, were identified, and the structural biological experiments showed that these inhibitors can bind to the pocket of PLpro of SARS-CoV-2, (Figure 1B; Table 1), providing a molecular basis for the substrate specificity and the inhibitory mechanisms (Rut et al., 2020; Patchett et al., 2021). Since SARS-CoV-2-PLpro has the similar catalytic preference and activity with SARS-CoV PLpro, the inhibitors that have been developed for SARS-CoV PLpro can be repurposed against SARS-CoV-2. Therefore, GRL0617, which was designed for SARS-CoV, showed inhibition of PLpro of SARS-CoV-2 (Ratia et al., 2008; Gao et al., 2021) (Table 1). Based on the structure of GRL0617, several new compounds were synthesized and are also capable of inhibiting SARS-CoV-2 viral replication in cells (Osipiuk et al., 2021).

**TABLE 1:** Protease enzyme and RdRP inhibitors at or after clinical trials.

Target	Drug name	Structure	Clinical status	References
main protease (Mpro)	Nirmatrelvir/ritonavir	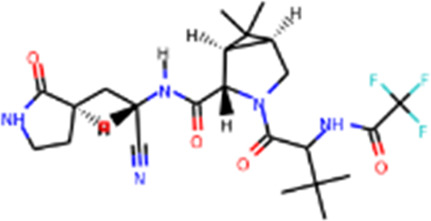 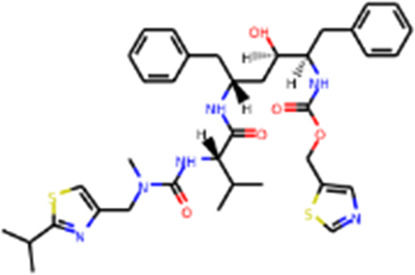	Emergency use authorization in COVID-19	Owen et al. (2021)
	Ebselen	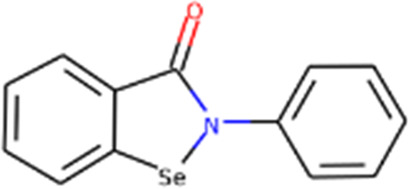	Phase 2 in COVID-19	Jin et al. (2020)
	Disulfiram	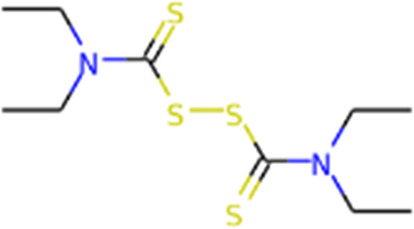	Phase 2 in COVID-19	Jin et al. (2020)
	PF-07304814	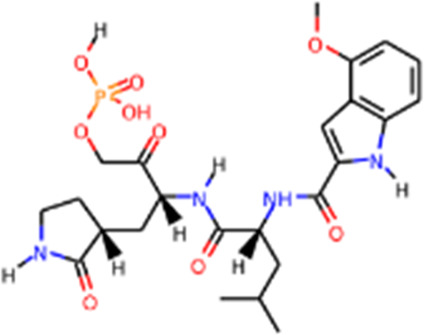	Phase 1 in COVID-19	Boras et al. (2021)
RNA-dependent RNA polymerase (Rdrp)	Remdesivir	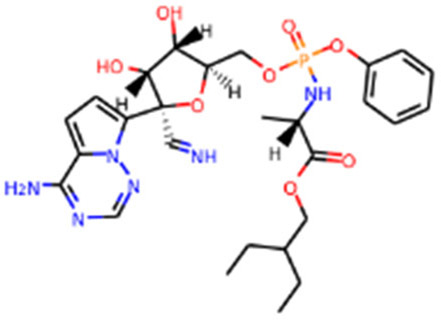	Emergency use authorization in COVID-19	Beigel et al. (2020)
	Molnupiravir	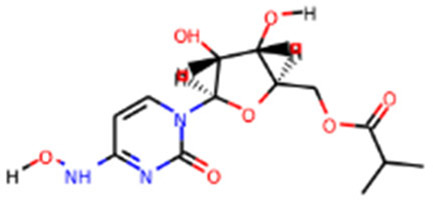	Emergency use authorization in COVID-19	Jayk Bernal et al. (2021)
	Favipiravir	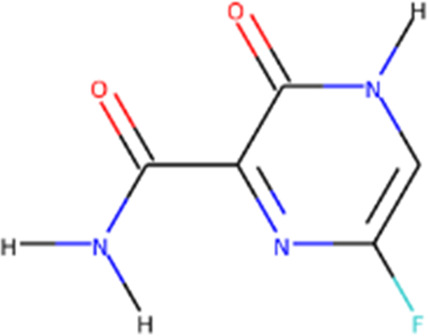	Phase 3 in COVID-19	Udwadia et al. (2021)
	Galidesivir	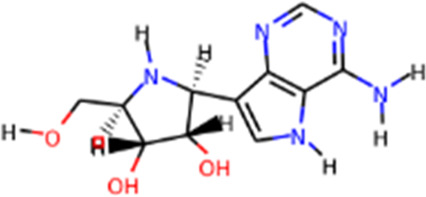	Phase 1 in COVID-19	Julander et al. (2021)
	Ribavirin	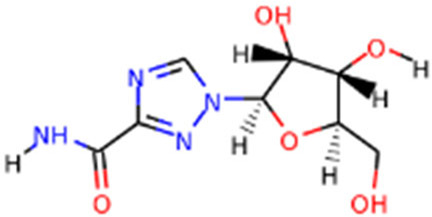	Phase 2 in COVID-19	Tong et al. (2020)
	Sofosbuvir	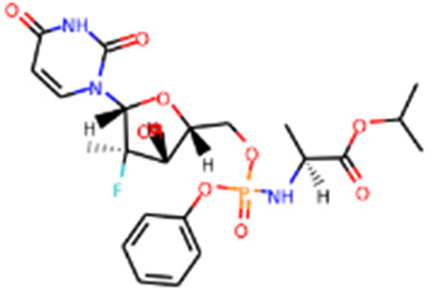	Phase 2 in COVID-19	Abbass et al. (2021)
	Emtricitabine/tenofovir disoproxil	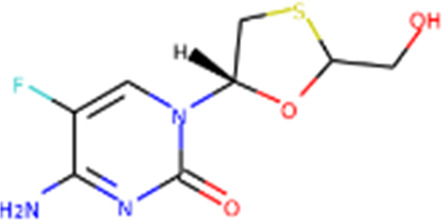 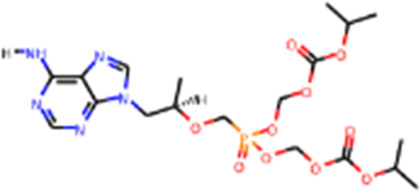	Phase 3 in COVID-19	DeJong et al. (2021)
	AT-527	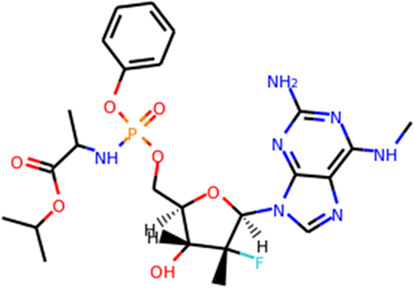	Phase 3 in COVID-19	Good et al. (2021)

#### Main proteases and inhibitors

Mpro, also called 3C-like protease (3CL protease), is another major protease encoded by SARS-CoV-2 RNA genome. Mpro is encoded by nsp5 (Figure 1A), and cleaves polyproteins, pp1a and pp1ab to release nonstructural proteins that mediate the assembly of the replication and transcription complex (Jin et al., 2020). The crystal structure of SARS-CoV-2 Mpro was reported soon after the identification of SARS-CoV-2 (Jin et al., 2020). Mpro contains three domains (Domains I, II, and III), which are conserved across coronaviruses. There is a substrate binding site under the gap of domain I and II, which has a catalytic dyad exerting proteolysis activity (Hegyi and Ziebuhr, 2002). In this dyad, Mpro forms a conserved binding pocket, and many drugs can bind to this site (Jin et al., 2020). In addition, Mpro is highly conserved and has no counterpart in host cells, which prompt targeting Mpro therapeutics will not induce the unnecessary side effect (Jin et al., 2020). Together, these make SARS-CoV-2 Mpro a promising virus-specific drug target.

Recently, An Emergency Use Authorization (EUA) was issued by Food and Drug Administration (FDA) for Paxlovid (Figure 1B and Table 1), developed by Pfizer, for the treatment of mild-to-moderate COVID-19. Paxlovid is comprised of nirmatrelvir, a Mpro inhibitor, co-packaged with ritonavir. Ritonavir is not the active ingredient to bind to Mpro, but functions as a regulator to prolong the duration of nirmatrelvir and increasing the drug plasma concentrations to inhibit SARS-CoV-2 replication (Anastassopoulou et al., 2022).

Significant efforts have been made to develop drugs targeting Mpro. Numerous inhibitors have exhibited binding activity to Mpro. Jin *et al* (Jin et al., 2020) identified several lead compounds targeting Mpro, including disulfiram, carmofur, ebselen, shikonin, tideglusib, PX-12, N3 and TDZD-8. Disulfiram can strongly inhibit Mpro with a half effective concentration (EC50) at 9.35 μM. And ebselen also exhibited a strong inhibition against Mpro. Both ebselen and disulfiram are under clinical trials for the patients with COVID-19 (NCT04485130 and NCT04484025). In addition, a computational docking analysis identified Bepridil, which displayed a huge potential for SARS-CoV-2 treatment in the *in vitro* assay. (Vatansever et al., 2021) (Table 1).

In addition, several studies reported a series of α-ketoamides that inhibit Mpro (Zhang et al., 2020). Another study presented two peptidomimetic aldehydes can inhibit Mpro activity through covalently binding to the C145 of the catalytic active center. Another two inhibitors labeled 11a and 11b, exhibited excellent inhibition of Mpro with low EC50 at 0.053 and 0.040 μM, respectively (Dai et al., 2020). Based on the strategy, there are a series of aldehyde derivatives that have been developed. MI-09 and MI-30, significantly reduced viral burden in the lungs of an *in vivo* model with good pharmacokinetic activity and safety in animals (Qiao et al., 2021). 6j was verified to reduce both SARS-CoV-2 infection in an *ex vivo* assay and the MERS-CoV viral titer of infected hDPP4-KI mice (Rathnayake et al., 2020). Guided by previous studies about Mpro of SARS-CoV, a panel of Mpro inhibitors reversibly bonding to the Mpro active-site cysteine C145 have been developed. Among these compounds, MPI5 and MPI8 could prevent SARS-CoV-2 infection in ACE2 expressing A549 and Vero cell lines. At the same time, MPI8 also showed high selectivity toward cathepsin L and high cellular and antiviral potency (Yang K. S. et al., 2021; Ma et al., 2022).

#### RNA-dependent RNA polymerase and inhibitors

Since SARS-CoV-2 is a positive sense RNA virus, RNA synthesis basing on RNA template is critical for SARS-CoV-2 viral transcription and replication (Naydenova et al., 2021). Nsp12, an RNA-directed RNA polymerase, is the key component of the replication and transcription complex (RTC) (Imbert et al., 2006). Nsp8 and nsp7 are two cofactors, which assist to the viral RNA transcription and production of viral particles in complex with nsp12 (Romano et al., 2020). There are three domains in the structure of SARS-CoV-2 RdRp, an interface domain connecting a core RdRp domain and a nidovirus-specific N-terminal domain (Gao et al., 2020). The core RdRp domain catalyzes RNA synthesis and maintains a relatively conserved architecture among the polymerase family of viruses (McDonald, 2013). It is comprised of three subunits, a finger, a palm and a thumb subdomain (Gao et al., 2020). The nidovirus-specific N-terminal domain contains a nidovirus RdRp-associated nucleotidyltransferase (NiRAN) structure. A new N-terminal b hairpin (residues D29 to K50) embeds into the pocket surrounded by the palm subdomain and NiRAN domain (Gao et al., 2020). The interface domain connects the right-hand RdRp domain and the NiRAN domain. The SARS-CoV-2 RdRp plays a similar role with cellular RNA polymerases and is also similar with SARS-CoV RdRp. These processes consist of RNA elongation, capping, and backtracking. (Dulin et al., 2015; Dulin et al., 2017; Chen J. et al., 2020; Malone et al., 2021). Another two nsps, nsp13 and nsp14, are also necessary to SARS-CoV-2 transcription and replication. The helicase nsp13 is an SF1B-family RNA helicase that could stably interact with the RTC of SARS-CoV-2. (Tanner et al., 2003; Ivanov et al., 2004; Lee et al., 2010; Malone et al., 2022). Nsp13 may participate in switching, backtracking, or disruption of the RNA genome template (Newman et al., 2021). Moreover, nsp14 could interact with RTC and play a role in proofreading activity. Since SARS-CoV-2 has a large RNA genome, replication fidelity is essential for maintaining genetic integrity. The backtracking function of nsp13 may assist nsp14 in approaching the mistake nucleotide and use its exonuclease activity to maintain high fidelity during RNA transcription and replication (Chen J. et al., 2020; Malone et al., 2021).

Since its essential role in RNA replication and its lack of a human homolog, RdRp is an important druggable target for anti-SARS-CoV-2 drug development. There are two types of RdRp inhibitors: nucleoside analogue inhibitors (Hall et al., 2021) and nonnucleoside analogue inhibitors (Yin et al., 2021). Remdesivir and molnupiravir are two nucleoside analogue inhibitors, which have been issued EUA by the U.S. FDA (Beigel et al., 2020; Jayk Bernal et al., 2021) (Table 1). Remdesivir is an adenosine analogue and once incorporated, will induce immediate pausing of RNA synthesis (Gordon C. J. et al., 2020). The structure of pre-translocated catalytic RTC incorporated with remdesivir clearly demonstrates its mechanism (Figure 1B). Unlike classic chain terminators, delayed chain termination occurs when remdesivir proceeds to the i+3 position. The incorporated remdesivir will be at position −3 or −4 for the pre- or post-translocated state, respectively (Wang Q. et al., 2020; Kokic et al., 2021). As a prodrug, molnupiravir can be converted into a cytidine analogue in the human body. The cytidine analogue exerts transition mutations during viral replication through indiscriminately incorporating either A or G (Kabinger et al., 2021). In addition, there are several other nucleoside analogue inhibitors, including galidesivir, favipiravir, Ribavirin, and AT-527, that are currently being evaluated in clinical trials (Table 1).

### Structural proteins as drug targets

The 3’ one-third RNA genome encodes S, M, E, N viral structural proteins. In addition to these structural proteins, the accessory genes are also located on this region. Although the function of accessory genes in SARS-CoV-2 are still not completely understood, some of them can modulate host innate or adaptive immune response (Nelson et al., 2020; Redondo et al., 2021). The most studied and first reported was the S protein, which mediates viral entry, providing basic information for the development of a neutralizing antibody.

#### S protein and S protein-neutralizing antibodies

**FIGURE 2:**
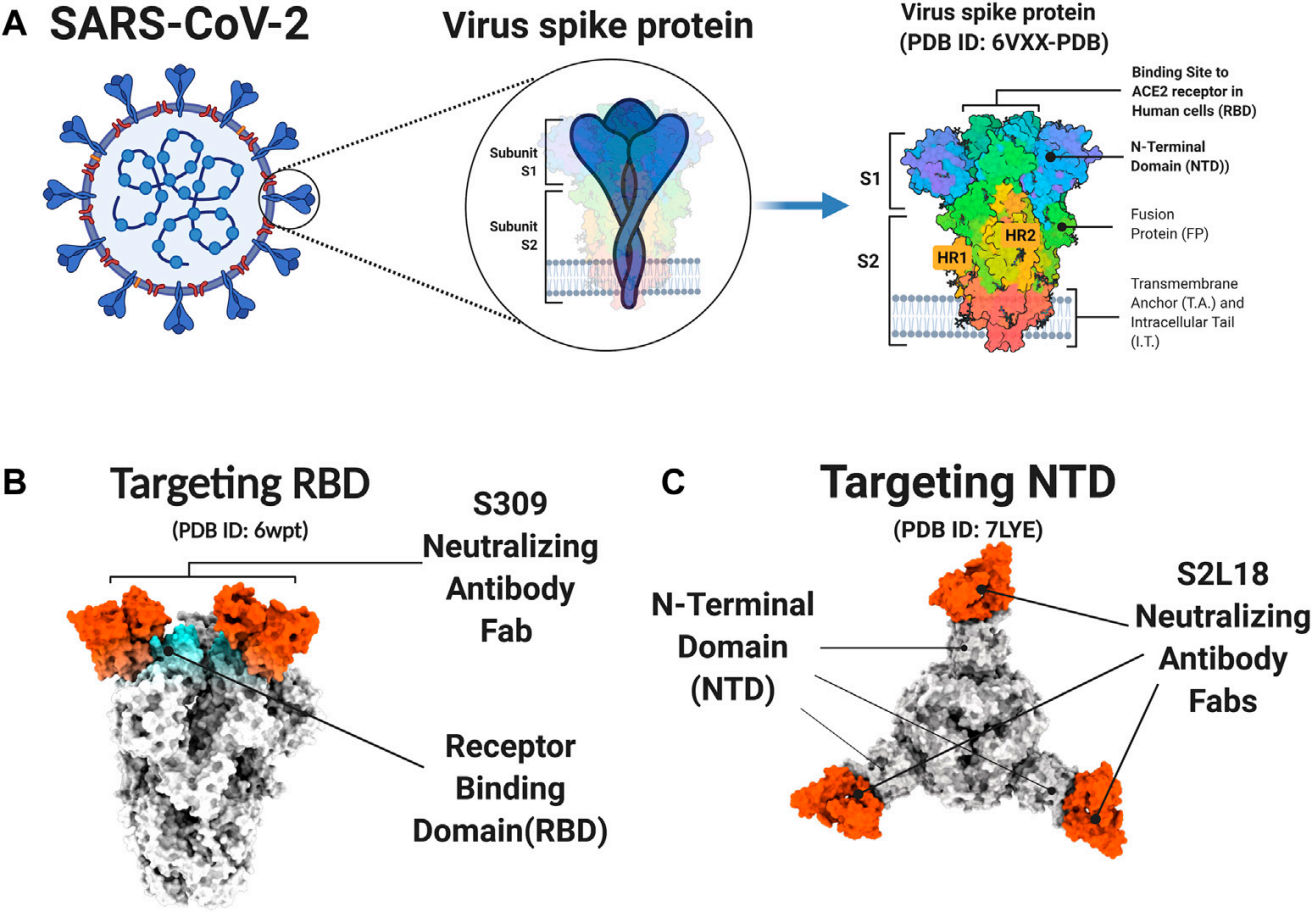
**S protein and neutralizing antibody. (A)** The Structure of the SARS-CoV2 S Glycoprotein. **(B-C)** The PDB model of S protein and neutralizing antibody. The S309 neutralizing antibody targets the receptor-binding domain (RBD) of S protein **(B)** The S2L18 neutralizing antibody targets the N-terminal domain (NTD) of S protein **(C)**. Figure was generated by BioRender.

S protein is a trimers structure that forms a crown on the surface of the viral particle (Figure 2A). It mediates virus entry and determines host tropism and pathogenesis. The S protein has two subdomains, S1 and S2, which are cleaved by furin or TMPRSS2 protease. S1 mediates binding to the receptor of host cells and S2 is responsible for membrane fusion (Walls et al., 2020). The S1 subunit contains two functional domains, the C-terminal domain (CTD) and the N-terminal domain (NTD). Part of the S1 subunit is a receptor-binding domain (RBD), which is the core domain and contains a receptor binding motif (Figures 2B,C). To bind the human angiotensin-converting enzyme 2 (hACE2) receptor, the RBD of S1 exhibits the up conformational movement, which enables RBD access to hACE2. When in the closed or “down” conformation, the RBD is hidden in the center (Wrapp et al., 2020). Due to the critical function of S protein, it is an attractive target of neutralizing antibodies (nAbs). The SARS-CoV-2 RBD is the main target of nAbs [6, 7]. Like the RBD, much of the S1 NTD is also exposed on the spike trimer surface and is targeted by neutralizing antibodies. The NTD plays a role in the conformation transition of the S protein. Many studies have proved that potent NTD antibodies confer protection against SARS-CoV-2 challenge, which highlights the importance of NTD-specific nAbs (Chi et al., 2020; McCallum et al., 2021).

Major effort has been devoted to leverage antibody therapeutics. Based on the targets on the SARS-CoV-2 S protein, the current antibody therapeutics can be classified into RBD targeted nAbs, NTD targeted nAbs and polyclonal antibodies. As shown in Table 2, there are several antibody therapeutics targeting RBD that have been granted emergency use authorization or are currently under clinical trials. Currently, four anti-SARS-CoV-2 nAb products or antibody cocktails have been issued EUAs by FDA, bamlanivimab plus etesevimab, casirivimab plus imdevimab, sotrovimab, and tixagevimab plus cilgavimab for the treatment of non-hospitalized mild to moderate patients with SARS-CoV-2 infection. However, according to the NIH COVID-19 Treatment Guidelines, the use of bamlanivimab plus etesevimab and casirivimab plus imdevimab have not been recommended, since the protective effect disappears against the B.1.1.529 variant (Cameroni et al., 2022; Cao et al., 2022; Liu et al., 2022). Sotrovimab, however, did exhibit antibody neutralizing activity against the Omicron variant in lab testing, and is therefore expected to retain its therapeutic efficacy against this variant. Moreover, another long-acting anti-SARS-CoV-2 mAb combination, tixagevimab plus cilgavimab, also maintained its effectiveness for neutralizing the Omicron variant. (Cameroni et al., 2022; Li et al., 2022; Liu et al., 2022). The nAbs under phase three trials still face the challenges of Omicron variant escape. Therefore, nAbs interventions must be adjusted in real time according to the evolutionary trajectory of SARS-CoV-2. (Table 2).

**TABLE 2:** Antibody treatments for COVID-19 at or after phase three clinical trial.

Target	Drug name	PDB ID	Clinical status	References
receptor-binding domain (RBD)	REGN-COV (REGN10933/casirivimab + REGN10987/imdevimab)	6XDG	Emergency use authorization in COVID-19	Weinreich et al. (2021)
	Bamlanivimab (LY3819253 or LY-CoV555) + etesevimab (LY3832479, LY-CoV016)	7KMG + 7F7E	Emergency use authorization in COVID-19	Tuccori et al. (2021)
	Sotrovimab (VIR-7831/GSK4182136)	7TLY	Emergency use authorization in COVID-19	Gupta et al. (2021)
	AZD7442 (AZD8895/tixagevimab + AZD1061/cilgavimab)	7L7E	Emergency use authorization in COVID-19	Mahase, (2021)
	Regdanvimab (CT-P59)	7CM4	Approved by European Commission and South Korea	(Lee et al., 2021; Syed, 2021)
	TY027	Not available	Phase 3	Kreuzberger et al. (2021)
	Amubarvimab + romlusevimab (BRII-196 + BRII-198)	Not available	Phase 3	Group, A.C.-T.f.I.w.C.-S. et al. (2021)
	Etesevimab (JS016, LY-CoV016, LY3832479)	7F7E	Phase 3	Kreuzberger et al. (2021)
	DZIF-10c, BI 767551	6XDG	Phase 2/3	Halwe et al. (2021)

## Drug development against host factors

### SARS-CoV-2 receptors

**FIGURE 3:**
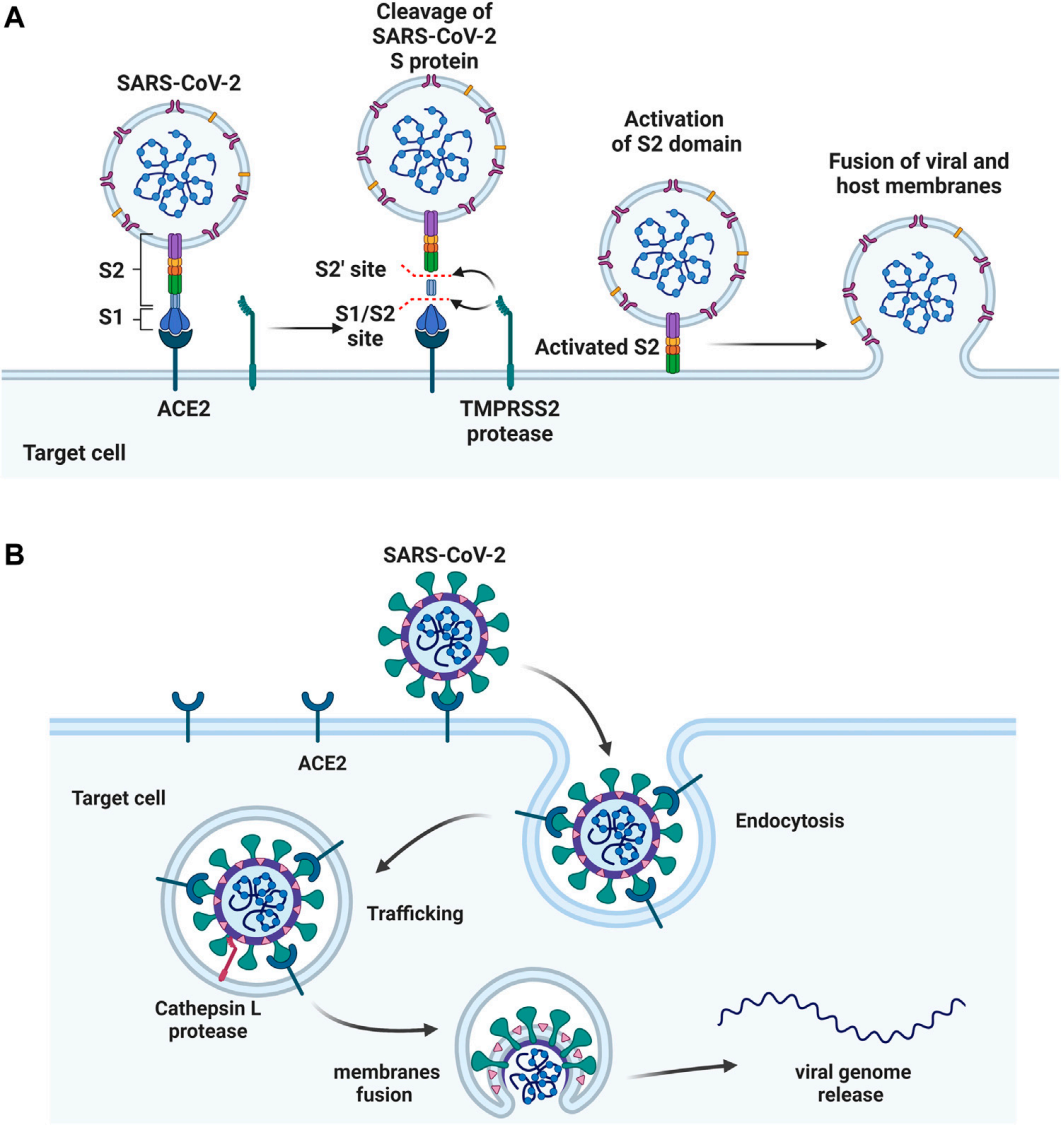
**Two approaches of SARS-CoV-2 entry. (A)** S protein binds to the host ACE2 receptor, followed by cleaving at S1/S2 and S2’ sites by TMPRSS2 protease. S2 domain mediates the fusion of viral and host membranes. **(B)** The S protein binds to ACE2, followed by the receptor mediated endocytosis. The viral fusion occurs in cytoplasm after S activation by host protease cathepsin L. Figure was generated by BioRender.

As a major receptor of SARS-CoV-2 (Shang et al., 2020a; Hoffmann et al., 2020b), ACE2 is a carboxypeptidase that removes a single amino acid from the C terminus of angiotensin I to angiotensin II, which are generated by renin and ACE (Gheblawi et al., 2020). Protein structural analysis revealed that the peptidase domain of ACE2 could bind to the receptor binding motif of the SARS-CoV-2 S protein (Lan et al., 2020; Yan et al., 2020) (Figure 3A). Lack of ACE2 blocks SARS-CoV-2 infection in Huh7.5 (Hoffmann et al., 2021a; Schneider et al., 2021) and Caco-2 (Gordon et al., 2020b) cells. In addition to ACE2, two groups reported neuropilin1 (NRP1) as another entry factor for SARS-CoV-2 (Cantuti-Castelvetri et al., 2020; Daly et al., 2020). TMPRSS2-mediated entry of wild-type SARS-CoV-2 could be enhanced by the presence of NRP1. Mutations at the furin cleavage site could decrease viral infection, which provides evidence that NRP1 requires a furin-cleaved substrate for its function (Cantuti-Castelvetri et al., 2020).

Multiple efforts have been pursued to exploit ACE2 as a therapeutic target. The fundamental principle of targeting ACE2 is to block the accessibility of virus to membrane-bound ACE2. The use of soluble ACE2 as a decoy receptor capable of trapping the virus to prevent membrane attachment is under investigation (Monteil et al., 2020; Zoufaly et al., 2020). Our study suggested that both imatinib and quinacrine dihydrochloride can bind with ACE2 and block the viral entry (Han et al., 2021). Other strategies target ACE2 using pseudoligands, blocking antibodies, or inhibitors downregulating ACE2 expression. Isotretinoin downregulates ACE2 expression (Sinha et al., 2020), and it also reduces dihydrotestosterone levels and downregulates TMPRSS2 (Table 3). Notably, ACE2 is a key enzyme of the renin–angiotensin–aldosterone system (RAAS), which is a commonly prescribed hypertension drug target. Studies suggested that they increase ACE2 expression (Ferrario et al., 2005) and may therefore worsen COVID-19 severity. However, clinical investigations revealed no adverse effects (Cohen et al., 2021; Williams, 2021). (Table 3). For the alternative receptors of SARS-CoV-2, corresponding therapeutics were also studied for reducing the burden of COVID-19. Meplazumab, which blocks the entry of SARS-CoV-2 by targeting CD147, was tested in a phase II clinical trial (NCT04275245) in China (Masre et al., 2021). Moreover, a potential monoclonal neutralizing antibody against NRP1 is currently under investigation for SARS-CoV-2 infection.

**TABLE 3:** Inhibitors targeting host factors at or after phase three trials.

Target	Drug name	Structure	Clinical status	References
ACE2	Isotretinoin	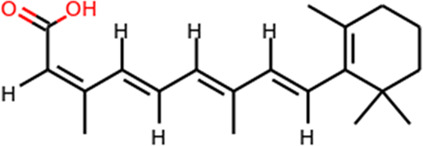	Phase 3	Demirel Ogut et al. (2021)
	Angiotensin Receptor Blockers	Not available	Phase 4	Hockham et al. (2021)
	Ensovibep	Not available	Phase 3	Tao et al. (2021)
TMPRSS2	Camostat Mesylate	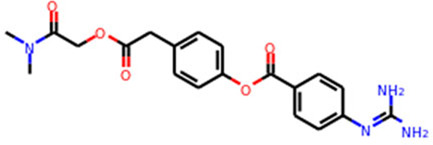	Phase 3	Hoffmann M. et al. (2021)
	Nafamostat Mesilate	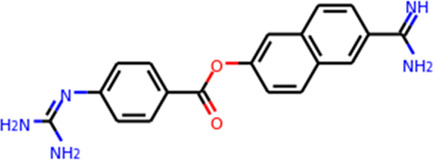	Phase 3	Zhuravel et al. (2021)
	Bromhexine	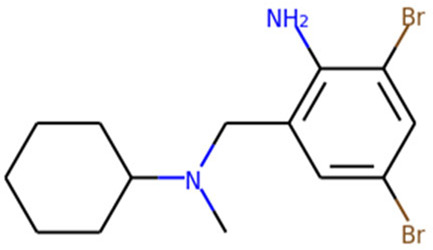	Phase 3	Ansarin et al. (2020)
	Bicalutamide	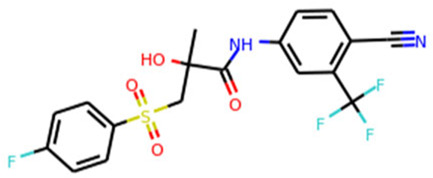	Phase 3	Welen et al. (2022)
Plasmin	Tranexamic acid	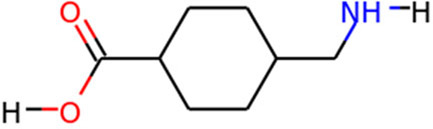	Phase 3	Ogawa and Asakura, (2020)
EEF1A1	Plitidepsin	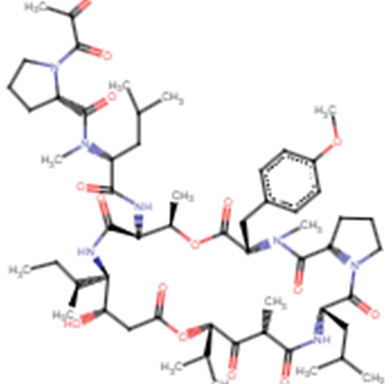	Phase 3	Varona et al. (2022)
tyrosine kinase	Fostamatinib	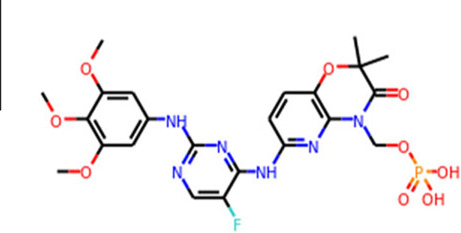	Phase 3	Strich et al. (2021)
SIGMAR1	Fluvoxamine	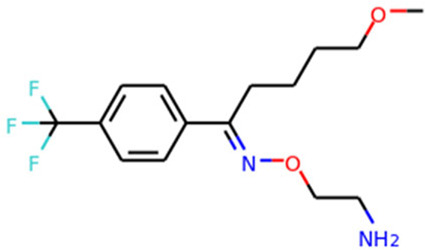	Phase 3	Reis et al. (2022)
ATP2C1	Sevoflurane	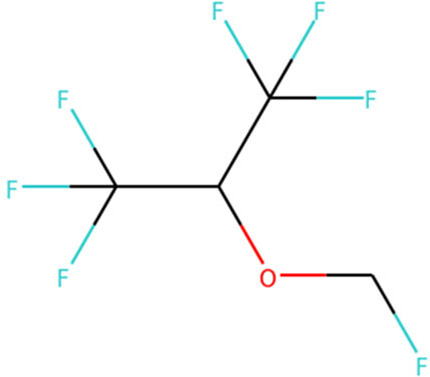	Phase 3	Jabaudon et al. (2017)
	Isoflurane	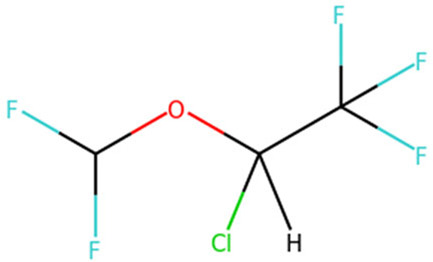	Phase 3	Flinspach et al. (2020)

### Proteases essential for SARS-CoV-2 entry

After binding, the S protein conformational transition depends on proteolytic cleavage, which depends on the types of target cell proteases. There are two cleavage sites, one is the S1/S2 boundary and the other is S2’ site of S2 subunit. For SARS-CoV-2, the S1/S2 boundary is cleaved by furin, while the S2’ site cleavage requires proteases in targeted cells. The other two proteases, TMPRSS2 and cathepsin L, activate S protein at different location of the cells. TMPRSS2-mediated S protein activation occurs at the cell surface (Figure 3A), whereas cathepsin-mediated activation occurs in the endo-lysosome (Figure 3B) (Hoffmann et al., 2020a; Sungnak et al., 2020; Zang et al., 2020).

Significant efforts have been applied to develop candidates targeting TMPRSS2 mediated S activation. The TMPRSS2 inhibitors camostat mesylate (Hoffmann M. et al., 2021), namostat (Jang and Rhee, 2020) and aprotinin (Bojkova et al., 2020) are being investigated in multiple ongoing clinical trials. As androgens are involved in the TMPRSS2 expression (Clinckemalie et al., 2013), several studies also investigated androgen-directed therapy (Bhowmick et al., 2020) (Table 3).

Additional work has been applied to develop drug candidates targeting cathepsins, endosomal cysteine proteases required for pH-dependent endocytic entry (Yang and Shen, 2020). Chloroquine and hydroxychloroquine, which inhibit the activity of cathepsin L, have proven antiviral activity in cell culture (Liu et al., 2020). However, chloroquine and hydroxychloroquine failed to block SARS-CoV-2 infection in an *in vivo* assay (Kaptein et al., 2020; Park et al., 2020) (Table 3). The clinical outcome of chloroquine and hydroxychloroquine is disappointing. Based on RECOVERY and WHO SOLIDARITY, two highly pragmatic trials, hydroxychloroquine treatment may lead to increased mortality in patients with COVID-19 (Axfors et al., 2021).

### Lipid and SARS-CoV-2 infection

Genetic screens have identified that several genes controlling fatty acid and cholesterol synthesis are involved in SARS-CoV-2 assembly and replication (Gordon et al., 2020a; Baggen et al., 2021; Chu et al., 2021; Schneider et al., 2021; Wang et al., 2021). Two P4-ATPase complex factors, TMEM30 A and ATP8B1, can transport aminophospholipids and mediate the membrane communication between the ER and other membranes, which were related with viral replication. Sterol-regulatory-element-binding proteins (SREBPs) are the key transcription factors that regulate fatty acid and cholesterol synthesis, which were identified in several genetic screens (Schneider et al., 2021; Wang et al., 2021). Our recent work reported that the compounds inhibiting fatty acid biosynthesis can block SARS-CoV-2 infection (Duan et al., 2021). This suggests Fatty Acid synthase and Acetyl-CoA carboxylase Alpha are potential host targets for drug development, which were verified using animal studies (Chu et al., 2021; Duan et al., 2021). 25-hydrocholesterol could inhibit S protein mediated membrane fusion of SARS-CoV-2 by consuming cholesterol from the cell plasma membrane. (Wang S. et al., 2020). Moreover, TMEM41B is identified as another critical host factor required for infection of human coronaviruses SARS-CoV-2 and SARS-CoV (Baggen et al., 2021; Hoffmann et al., 2021b). Together, this suggests that cholesterol and other lipids participate in the viral life cycle, including viral entry, intracellular transport, and replication complex formation. Viruses interact with lipid membranes to infect a cell and reprogram lipid metabolism to fuel replication.

### Potential therapeutic regimens targeting immune system and cytokine storm

**FIGURE 4:**
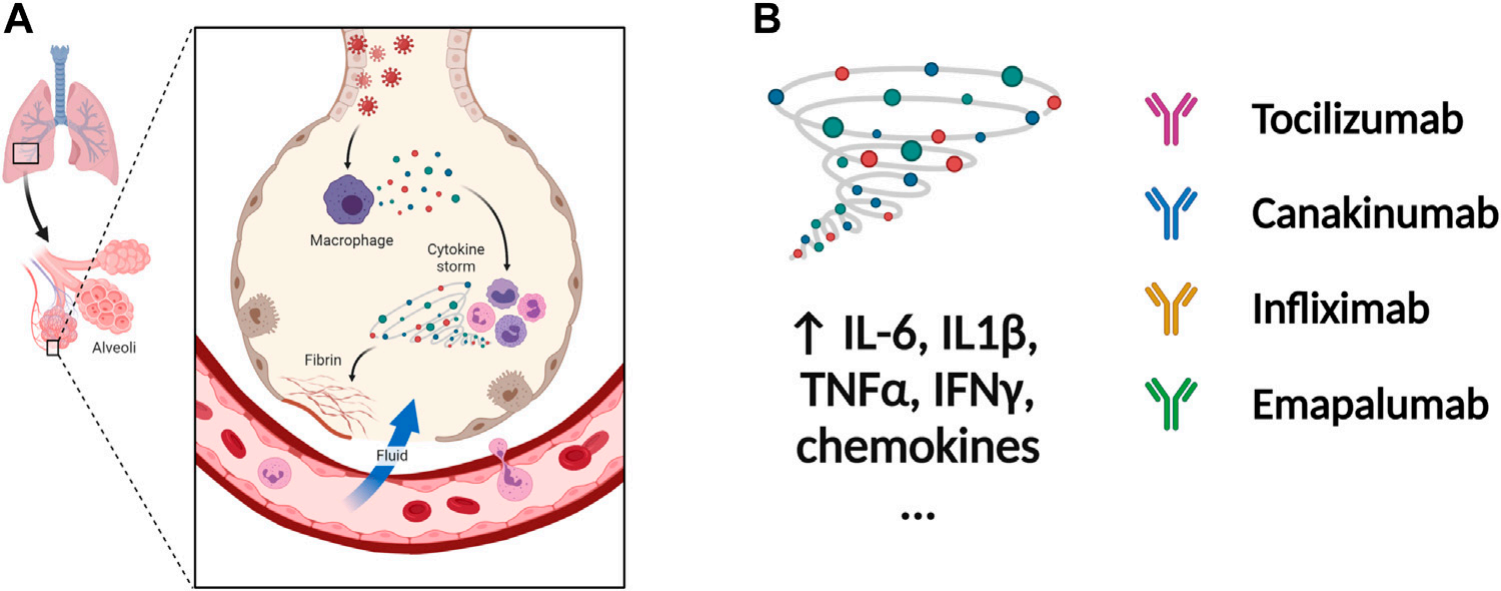
**Potential therapeutic regimens targeting the immune system and cytokine storm. (A)** The SARS-CoV-2 infected cells will secrete great amounts of chemokines or cytokines, which recruit immune cells including macrophages. The cytokine storm is caused by uncontrolled immune response that immune cells continuously activate, expand, and produce immense amounts of cytokines. The inflammation will damage the lung cells followed by the formation of fibrin and accumulation of fluid seeping into the lung cavities, leading to the failure of gas exchange. **(B)** The nAbs targeting IL-1β, IL-6, TNF-α, and IFN-γ among others have been tested in patients with severe COVID-19. inflammation. Figure was generated by BioRender.

The severity and modality of COVID-19 patients correlates with increased concentrations of circulating cytokines (de la Rica et al., 2020; De Virgiliis and Di Giovanni, 2020). The deaths of patients with COVID-19 are closely related with ARDS, which is often caused by an uncontrolled immune response. Continuous expansion and activation of inflammation and release of large amounts of inflammatory cytokines are the main characteristics of ARDS. (Del Valle et al., 2020). Recent cohort studies showed high circulating cytokine levels in patients with COVID-19 (Laing et al., 2020). Therefore, lowering the inflammatory response may be a potential therapeutic strategy for severe COVID-19. Anti-IL-6 receptor nAbs, such as sarilumab, tocilizumab, levilimab and anti-IL-6 mAb siltuximab, have been evaluated in several clinical (Zhou F. et al., 2020; Wang Z. et al., 2020; Huang et al., 2020) (Figures 4A,B and Table 4). An EUA of Actemra (tocilizumab) was issued by the FDA to treat the severe COVID-19 adults and children (more than 2 years old) patients. Moreover, other cytokines and growth factors, including TNF-α, IL-1β, granulocyte-macrophage CSF, vascular endothelial growth factor, and IFN-γ, among others, were exploited as potential drug targets for COVID-19 patients. Our recent studies using an immune-cardiac co-culture platform identified a JAK inhibitor that blocks macrophage-mediated inflammation and myocardial injury (Yang L. et al., 2021). The FDA also issued an EUA for baricitinib (Olumiant), a JAK inhibitor, in combination with remdesivir for confirmed COVID-19 patients. Glucocorticoids are widely used for repressing inflammatory reactions, so theoretically glucocorticoids can reduce the progression to respiratory failure and death in patients with COVID-19. Although the use of glucocorticoids was debated at the beginning of the pandemic (Shang L. et al., 2020; Russell et al., 2020), a randomized clinical trial of dexamethasone reported that patients with severe COVID-19 can benefit from the use of dexamethasone, which could lower the 28-days mortality in patients with invasive mechanical ventilation or oxygenation alone (Group et al., 2021).

**TABLE 4:** Inhibitors targeting the immune system and cytokine storm at or after phase three trials.

Target	Drug name	PDB or structure	Clinical status	References
C5a	IFX-1 (BDB-001)	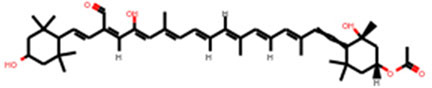	Phase 3	Vlaar et al. (2020)
CCR5	Leronlimab (PRO-140)	Not available	Phase 3	Agresti et al. (2021)
Connective tissue growth factor	Pamrevlumab (FG-3019)	Not available	Phase 3	Richeldi et al. (2020)
DAMPs, Siglec G/10	CD24Fc (SACCOVID)	Not available	Phase 3	Song et al. (2022)
GM-CSF	Lenzilumab	Not available	Phase 3	Temesgen et al. (2021)
GM-CSF receptor	Mavrilimumab	Not available	Phase 3	Cremer et al. (2021)
IFN gamma	Emapalumab (Gamifant)	Not available	Phase 3	Cure et al. (2021)
IL-1	RPH-104	Not available	Phase 3	Valenzuela-Almada et al. (2021)
IL-1 β	Canakinumab	5BVJ	Phase 3	Caricchio et al. (2021)
IL-6	Siltuximab	Not available	Phase 3	Gritti et al. (2021)
IL-6 VEGF	Olokizumab	4CNI	Phase 3	Antonov et al. (2020)
	Clazakizumab	Not available	Phase 3	Vaidya et al. (2020)
	Bevacizumab	7V5N	Phase 3	Pang et al. (2021)
C5	Ravulizumab-cwvz	Not available	Phase 3	McEneny-King et al. (2021)
IL-6R	Tocilizumab	Not available	Emergency use authorization in COVID-19	Rosas et al. (2021)
IL-6R CD6	Sarilumab (SAR153191, Kevzara)	Not available	Phase 3	Investigators et al. (2021)
	Levilimab (BCD-089)	Not available	Approved in Russia	Lomakin et al. (2021)
	Itolizumab (EQ001, H-T1, T1-h)	Not available	Approved in India and Cuba	Atal et al. (2020)

### Prospective

In the last two and half years, SARS-CoV-2 continues to evolve. Recently, the omicron variant has developed into BA.1, BA.2, and BA.3.1 variants (Viana et al., 2022). Omicron variant BA.2 has become the dominant strain in many places (Chen and Wei, 2022). The BA.1 variant has shown substantial escape from neutralizing antibodies induced by vaccination (Cele et al., 2022; Liu et al., 2022; Schmidt et al., 2022). A recent study estimated that BA.2 is about 1.5 times as contagious as BA.1, and 30% more capable than BA.1 to escape current vaccines (Chen and Wei, 2022). Overall, there is an urgent need to develop pan-effective antiviral drugs and nAbs. Here, we have summarized druggable targets and therapeutic development for SARS-CoV-2 infection. The potential treatment targets can be divided into two groups, essential viral proteins and host factors supporting the viral life cycle. The non-structural proteins, Mpro, PLpro, and RdRp, are attractive drug targets, since they play pivotal roles in mediating viral replication and transcription. The structural S protein, which mediates viral entry, is the main target of nAbs and vaccines. S protein is also a very challenging target due to the high variability that enhances immune escape. It is still challenging for the nAbs and vaccine development to keep up with the continuing viral mutations. Regarding host factors, ACE2, TMPRSS2, and cathepsins, which have critical roles in viral binding and membrane fusion, are promising drug targets to develop pan-inhibitors of SARS-CoV-2. A majority of genetic and drug screening, as well as protein interactome studies, have identified several host factors that play roles in viral replication, viral translocation, and assembly. Finally, COVID-19 can produce a systemic inflammatory reaction involving many organs, which is highly associated with the severity of the disease. Thus, blocking immune cell-mediated host damage and cytokine storm are also critical for anti-SARS-CoV-2 drug development. In summary, significant efforts have been applied to understand SARS-CoV-2, which has provided insights into novel anti-SARS-CoV-2 drug development.
